# NBCe2 (*Slc4a5)* Is Expressed in the Renal Connecting Tubules and Cortical Collecting Ducts and Mediates Base Extrusion

**DOI:** 10.3389/fphys.2020.00560

**Published:** 2020-05-29

**Authors:** Dagne Barbuskaite, Fredrik D. Pedersen, Henriette L. Christensen, Laura Ø. Johnsen, Jeppe Praetorius, Helle H. Damkier

**Affiliations:** ^1^Department of Cellular and Molecular Medicine, Faculty of Health and Medical Sciences, University of Copenhagen, Copenhagen, Denmark; ^2^Department of Biomedicine, Health, Aarhus University, Aarhus, Denmark

**Keywords:** NBCe2, kidney, blood pressure, *Slc4a5*, hypertension

## Abstract

Arterial hypertension, is a common disorder with multiple and variable etiologies. Single nucleotide polymorphism analyses have detected an association between variants in the gene encoding the electrogenic Na^+^:HCO_3_^–^ cotransporter NBCe2 (*Slc4a5*), and salt-sensitive hypertension. Mice with genetic deletion of NBCe2 are hypertensive, and the cause of the blood pressure (BP) increase is believed to arise from a lack of renal NBCe2 function. The exact cellular expression of NBCe2 in the kidney tubular system is, however, not determined. Here, we find NBCe2 to be expressed predominantly in isolated connecting tubules (CNT) and cortical collecting ducts (CD) by RT-PCR. In isolated renal CNT and CCD, genetic deletion of NBCe2 leads to decreased net base extrusion. To determine the role of renal NBCe2 in the development of hypertension, we generated CNT and intercalated cell NBCe2 knockout mice by crossing an *Slc4a5* lox mouse with mice expressing cre recombinase under the V-ATPase B1 subunit promotor. Although the mice displayed changes in the expression of renal membrane transporters, we did not detect hypertension in these mice by tail cuff recordings. In conclusion, while global NBCe2 deletion certainly causes hypertension this study cannot confirm the role of renal NBCe2 expression in blood pressure regulation.

## Introduction

Hypertension, defined as a chronically elevated blood pressure (BP), is one of the most common chronic disorders in the developed world, affecting nearly one billion people worldwide (Writing Group Members et al., 2016). Hypertension has a significant heritability factor, as more and more genes are being identified as hypertension susceptibility genes. *Slc4a5*, encoding the sodium bicarbonate cotransporter NBCe2, was shown to be significantly associated with BP increases (Barkley et al., 2004; Hunt et al., 2006), and a salt-sensitivity trait (Carey et al., 2012; Taylor et al., 2012) by several genome wide association studies. Studies of NBCe2 knockout (NBCe2 ko) mice confirmed this observation. In the pioneering study by Groger et al. (2012) an NBCe2 ko was generated by excising the 7th coding exon, which resulted in increased BP at day and night time, metabolic acidosis, increased arterial natriuretic peptide, hyporeninemia, hypoaldosteronism, and increased glomerular filtration rate. A quite different phenotype was observed in the studies by Wen et al. (2015b) in the same ko model, but backcrossed to a C57Bl/6 background for at least 6 generations, resulting in a BP phenotype only on an acidic diet. The authors showed that NBCe2 dysfunction leads to increased activity of epithelial sodium channels (ENaC) in the kidney, as well as increased uncleaved α-ENaC expression and decreased NCC expression (Iversen et al., 2013). In the latter study of NBCe2 dysfunction, NBCe2 ko was reported to cause distal renal tubular acidosis and increased H^+^-ATPase expression. Additionally, the two studies by Wen et al. report different results on the same NBCe2 ko mouse model: in the first study, decreased urinary (Na^+^), and increased (K^+^) excretion was reported and blood (Na^+^) and (K^+^) was unchanged, whereas the authors report decreased blood (K^+^), and increased blood (Na^+^), with no changes in urine concentration in the second study. The explanation for the observed NBCe2 ko phenotype was also different: Gröger et al. reasoned that lack of NBCe2, that supposedly reabsorbs bicarbonate in the collecting duct (CD), causes upregulation of another sodium bicarbonate transporter (NBCn1). Due to different transport stoichiometry increased activity of NBCn1 results in increased sodium reabsorption. Wen et al. suggested a different role for NBCe2. They propose NBCe2 to be expressed on the basolateral membrane in the principal cells and have a role in intracellular pH regulation, thus having most activity during acidosis, and causing intracellular sodium loading and inhibition of luminal ENaC activity. Such different interpretation of the results arises from overall lack of information about the exact renal NBCe2 expression site.

NBCe2 mediates electrogenic Na^+^:HCO_3_^–^ transport (Sassani et al., 2002), and can operate in both a 1:2 (Virkki et al., 2002; Shao et al., 2009), and 1:3 (Pushkin et al., 2000; Millar and Brown, 2008) manner, but the transport stoichiometry as well as directionality have not been defined for the renal NBCe2. In addition, there is a lack of coherence regarding NBCe2 localization in the kidney. The expression of NBCe2 has been reported in nearly all kidney nephron segments and CDs, indicating that some species-specificity might be present. In humans, NBCe2 has been immunolocalized in the intercalated cells of the CD (Damkier et al., 2007), and in the proximal tubules (PT; Gildea et al., 2015). In isolated rat tubules, NBCe2 mRNA expression was detected in cortical thick ascending limb (cTAL), medullary thick ascending limb (mTAL), PT, and cortical CD (Xu et al., 2003) but the purity of the micro-isolated renal tubules was not reported. In mouse, *in situ* hybridization studies showed that NBCe2 co-localizes with AQP2, which indicates expression in the principal cells of the CD (Groger et al., 2012). NBCe2 was also detected in micro-isolated connecting tubules (CNT; Wen et al., 2015a), again without reporting how the purity of the samples was tested. Finally, a transcriptome study evaluating renal CD cells revealed that *Slc4a5* expression was detected only in a few of the α- and β-intercalated cell (2/87 and 1/23, respectively) as well as principal cell (2/74) samples (Chen et al., 2017). Thus, it is complicated to establish a physiological and pathophysiological role for NBCe2 in the kidney without knowledge on which tubule segments express the protein, what membrane domain it localizes to, and finally the transport direction of NBCe2 at these sites.

In the current study, we evaluate the NBCe2 expression in isolated renal tubules and detect expression only in cells from CNT and CCD. Using intracellular pH recordings of isolated CNT and CDs, we determine that global lack of NBCe2 expression leads to impaired base extrusion capacity. We find decreased expression of γ-ENaC and increased expression of NBCn1 in global NBCe2 ko mice. Finally, we crossed floxed NBCe2 mice with a mouse expressing cre recombinase driven by the V-ATPase B1 promotor. This *Slc4a5*^(lox/lox)cre(Atp6v1b1)^ mouse model showed decreased expression of α-ENaC but increased expression of γ-ENaC. Furthermore, the expression of the acid/base transporters AE2, V-ATPase, and pendrin were decreased in this model. Finally, tail cuff BP measurements confirmed hypertension in the global NBCe2 knockout mouse, but surprisingly the kidney-specific NBCe2 knockout mice did not display a BP phenotype. Our study suggests that a lack of NBCe2 expression in the CNT and CD does not seem to be associated with the hypertension observed in global NBCe2 knockouts. This suggests that another organ or cell type is involved in the BP regulation in the global NBCe2 knockout mouse.

## Materials and Methods

### Experimental Animals

All animal experiments and protocols were approved by the Danish Animal Experiments Inspectorate. Mice were fed a standard rodent pellet diet (0.4% sodium) *ad libitum*, had free access to tap water and were exposed to a 12 h:12 h light-dark cycle. Only male mice were used throughout the study.

Transgenic mice, expressing enhanced green fluorescent protein (eGFP) in particular renal tubule segments were used for kidney tubule isolation. Mice expressing eGFP under the parvalbumin (PV-eGFP) promoter (Markadieu et al., 2012) were used for collection of distal convoluted tubules (DCT). Transgenic mice expressing eGFP driven by the transient receptor potential subfamily V, member 5 gene (TRPV5; Hofmeister et al., 2009) were used to collect late DCT, CNT, and CDs.

The breeding and genotyping of NBCe2 ko mice on a mixed C57Bl/6J-129S1/Sv genetic background have previously been described (Christensen et al., 2018). Briefly, the 13th exon of the *Slc4a5* gene was floxed with LoxP sites, and the generated *Slc4a5*^flx^ mice were crossed with a tamoxifen-inducible ubiquitin promoter-driven Cre-recombinase expressing mouse strain. Mice carrying the *Slc4a5*^flx^ allele were also used to generate kidney specific knockout mice. Intercalated cell specific kidney knockout mice were generated by crossing mice with the *Slc4a5*^flx^ allele, and mice expressing Cre recombinase under endogenous Atp6v1b1 promoter/enhancer elements (Miller et al., 2009) resulting in *Slc4a5*^(lox/lox)cre(Atp6v1b1)^. In the following, this strain will be named NBCe2^B1–VATPasecre^ mice. Throughout the study, mice carrying *Slc4a5*^(lox/lox)^ without cre expression or wildtype mice expressing only cre are used as controls and will be named “control.” In all experiments, littermates are used as controls in order to ensure similar genetic background when comparing phenotypes.

### NBCe2 Expression in Micro-Isolated Mouse Kidney Tubules and Cells

The kidney tubule isolation procedure was adapted from Hofmeister et al. (2009). Transgenic TRPv5-promoter driven eGFP expressing mice (CNT/CCD-GFP) and parvalbumin-promoter driven eGFP expressing mice (DCT-GFP) were anesthetized by isoflurane inhalation and perfused through the left ventricle with isolation buffer (in mM: 150 Na^+^, 3.6 K^+^, 1.0 Mg^2+^,1.3 Ca^2+^, 140 Cl^–^, 0.4 H_2_PO_4_^–^, 1.6 HPO_4_^2–^, 1.0 SO_4_^2–^, 10 acetate^–^, 1.3 gluconate^–^, 1.0 α-ketoglutarate, and 5 glycine) supplemented with 48 mg/l trypsin inhibitor, 25 mg/l DNase I, 1 mg/ml collagenase B (Roche Diagnostics), and 1 mg/ml pronase (Roche Diagnostics) at pH 7.4 and 37°C. Kidneys were dissected and placed in the enzyme-supplemented isolation buffer and spun at 850 rpm for 10 min. To isolate inner medullary CDs, 1 mg/ml Hyaluronidase was added. Half volume of the tubule suspension was transferred into ice-cold isolation buffer containing 0.5 mg/ml albumin. Equal volume of isolation solution was added to the remaining tubule suspension and spun for an additional 5 min. This procedure was repeated three times resulting in 4 tubule fractions. PT, thick ascending limbs of loop of Henle (TAL), and glomeruli (Gl) were selected under a dissection microscope (Leica MZ125) according to their morphology. DCT and CNT/CD were collected according to fluorescence. Neither the DCT nor CNT/CD collected fractions were pure, thus we determined NBCe2 by fluorescence activated cell sorting (FACS) DCT and CNT/CD tubules.

For FACS (Jensen et al., 2012), the isolation buffer was supplemented with 1 μl/ml proteinase K (Roche Diagnostics), and mice were perfused as described above. The perfused kidneys were dissected, sliced, and incubated in isolation solution for 45 min on a shaking table at 37°C. To obtain single cells, tubules were digested twice in a trypsin/ethylenediaminetetraacetic acid (EDTA) solution (Invitrogen, ThermoFisher, Roskilde, Denmark) at 37°C for 5 min. The cell suspension was passed through a 40 μm filter, and propidium iodide was added before the FACS for exclusion of dead cells. Tubules were sorted into fluorescein-positive and fluorescein-negative samples by 4-way purity sorting on a FACS Aria III (BD Biosciences, Lyngby, Denmark). The tubules were sorted using a 100 μm nozzle, at 20 psi and 30 kHz. For FACS on kidney cells from TRPv5-eGFP mice, 25,739,077 events were recorded, and 109,449 CNT/CCD-GFP positive (+) cells were collected with a recount purity of 94.5%, and 4,604,668 negative eGFP(–) cells were collected with 100.0% purity, respectively. For FACS on kidney cells from parvalbumin-eGFP mice, 21,309,632 events were recorded, and 105,884 DCT-GFP positive (+) cells were collected with a recount purity of 94.0%, and 3,914,983 negative eGFP (–) cells were collected with 99.7% purity, respectively. Collected tubule samples were placed in RNAlater solution (Invitrogen) for later analysis by RT-PCR.

### Reverse Transcription Polymerase Chain Reaction (RT-PCR)

The purity of the collected renal tubules and the expression of NBCe2 was determined by RT-PCR (see Supplementary Table S1 for primer sequences and product sizes). RNA from the tubules was purified using an RNeasy micro kit (Qiagen, Copenhagen, Denmark) according to the manufacturer’s instructions. The concentration of purified RNA was determined by absorbance at 260 nm using a NanoDrop ND-2000 (Fisher Scientific) and 20–40 ng of RNA was reverse transcribed by iScript Reverse Transcription Supermix (Bio Rad, Copenhagens, Denmark). PCR amplification was performed for each transcript by mixing cDNA with 1 pmol of primers and 5× HOT FIREPol Blend Master Mix (Solis BioDyne). The PCR reaction was performed for 35 cycles after 15 min at 95°C: denaturation was performed for 30 s at 95°C, annealing at 60°C for 30 s, and elongation at 72°C for 1 min. PCR products were mixed with 2 μl of DNA Gel Loading Dye (6×; Thermo Scientific) containing 0.05% 10000 × GelRed (Biotium, BioNordika Denmark, Herlev, Denmark) nucleic acid gel stain, separated by 1% agarose gel electrophoresis, and photographed under ultraviolet illumination.

### Intracellular pH Measurements in Distal Renal Tubules

Isolated renal tubules were resuspended in a HEPES-buffered solution (HBS; see Supplementary Table S2), plated on Cell-tak (corning^TM^, Fisher Scientific)-covered coverslips and allowed to attach for 10 min at 37°C. Cells were loaded with the pH-sensitive BCECF (10 μM, Thermo Fisher Scientific, Roskilde, Denmark) for 10 min or carboxy-SNARF (2 μM, Thermo Fisher Scientific) for 30 min. Coverslips were mounted in a closed perfusion chamber (RC-21BR; Harvard Apparatus, Holliston, MA, United States) and placed on an inverted microscope stage inside a 37°C dark chamber. Tubules were allowed to equilibrate in HBS before the protocols were performed. CNT/CCD segments were identified by branching appearance and the occurrence of intercalated cells not readily retaining the fluorescent dyes.

Intracellular pH with BCECF was recorded similar to a previous study (Hofmeister et al., 2012). In short, the tubules were imaged at the stage of a Nikon Eclipse microscope provided with a Nikon Plan Apo VC 60×/1.4NA oil-immersion objective. Till Vision software (Till Photonics) was used to control the monochromator wavelength (alternating between 490 nm and 440 nm), frequency (1 Hz), exposure time (20 ms), and binning (to 640 × 480 pixel images). A 12-bit cooled monochrome CCD camera (Imago, Till Photonics) recorded the light emission at 510–535 nm and data was collected from user defined regions of interest (ROIs) of individual tubule cells after background subtraction. Sample size (*n*) refers to the mean values from at least three individual cells from one mouse. In separate experiments the fluorescence ratio was calibrated to pH by clamping pH_i_ stepwise from pH 8 to 6 in a high K^+^ containing HBS with 10 μM nigericin (Sigma-Aldrich, Soeborg, Denmark) similar to (Boyarsky et al., 1988).

To determine acid-extrusion, the tubules were acidified by superfusion with a HEPES buffered solution containing 20 mM NH_4_Cl (HBS NH_4_Cl) for 3 min followed by a washout of NH_4_Cl using a Na^+^ free bicarbonate buffered solution (BBS 0Na^+^, see Supplementary Table S2). After reaching a new steady state, a Na^+^ containing bicarbonate solution was added (BBS, Supplementary Table S2) and the Na^+^ dependent pH recovery was determined as the dpH_i_/dt after re-addition of Na^+^.

For pH_i_ recordings using carboxy-SNARF, the tubules were imaged using an iMic microscope (Till Photonics) with an Olympus UApo N340, 40×/1.35 NA oil-immersion objective. Till Vision software (Till Photonics) was used to control the monochromator wavelength for excitation alternating between 485 nm and 555 nm, exposure time (25 ms), frequency (0.25 Hz), and binning (to 256 × 256 pixel images). Light emission (565–615 nm) was recorded by a 14-bit cooled monochrome EMCCD camera (iXon^EM+^, Andor technology, Belfast, United Kingdom) with 4 times EM gain. Data was collected from ROIs of individual tubule cells after background subtraction. The excitation fluorescence ratio (485/555 nm) was calibrated to pHi by stepwise shifts from pH 8.4 to 7 in high K^+^ containing HBS with 10 μM nigericin.

To determine base extrusion, the tubules were superfused with a bicarbonate buffered solution (BBS, Supplementary Table S2). Base extrusion was determined as the dpH_i_/dt following peak alkalization induced by removal of Cl^–^ in a bicarbonate buffered solution (BBS 0Cl, Supplementary Table S2).

The intrinsic buffering capacity was determined by recording of pH_i_ changes while stepwise decreasing NH_4_^+^ from 20 to 0 mM as previously described (Boyarsky et al., 1988; Hofmeister et al., 2012).

All experiments were performed in the presence of 2.5 mM probenecid (Thermo Fisher Scientific) to inhibit dye extrusion by organic anion-transporters. CNT/CD tubules were selected based on morphology similar to a previous study (Hofmeister et al., 2012).

### Immunoblotting

Mouse kidney was homogenized in an ice-cold dissection buffer (0.3 mol/L sucrose, 25 mol/L imidazole, 1 mmol/L EDTA, and pH 7.2) containing 8.4 mol/L leupeptin (Calbiochem) and 0.4 mmol/L Pefabloc (Roche) protease inhibitors, as well as PhosSTOP (Roche, Sigma Aldrich, Soeborg, Denmark) phosphatase inhibitor (1 tablet/10 ml buffer). Samples were centrifuged at 4000 *g* for 15 min at 4°C, and the sample buffer was added to the supernatant (0.1 mol/L sodium dodecyl sulfate and 0.04 mol/L dithiothreitol, pH 6.8). The protein samples were heated at 65°C for 15 min and separated by 4–15% gradient polyacrylamide gel electrophoresis (Bio Rad, mini-protean TGX). Then samples were electro transferred by the Transblot turbo system (Bio Rad) onto a PVDF (Ambion, ThermoFisher, Roskilde, Denmark) membrane, which was then blocked with 5% milk in PBS-T (in mmol/L: 167 Na^+^, 2.8 H_2_PO_4_^–^, 7.2 HPO_4_^2–^, and pH 7.4 with 0.1% vol/vol Tween), and incubated with primary antibody in PBS with 1% bovine serum albumin (BSA), and 2 mmol/L NaN_3_ overnight at 4°C. The primary antibodies used in the study were characterized elsewhere: NBCe2 (Christensen et al., 2018), α-ENaC (Sorensen et al., 2013), the 82 kDa band represents uncleaved and the 25 kDa band cleaved α-ENaC (Michlig et al., 2005), γ-ENaC (Masilamani et al., 1999) [the uncleaved form is seen at 95 kDa and the cleaved form at 65 kDa (Harris et al., 2008)], and H^+^-ATPase (Toyomura et al., 2000). The day after, membranes were rinsed, and incubated with horseradish peroxidase-conjugated anti-rabbit secondary antibody (Dako) diluted 1:3000 in 5% milk in PBS-T at room temperature. The membranes were incubated with the Pierce ECL Plus Western Blotting Substrate (Thermo Scientific) and imaged with the Epson perfection V700 Photo scanner (Seiko Epson Corporation, Suwa, Japan). Labeling density was quantified using Quantity One 4.6.9 software (Bio Rad Laboratories).

### Metabolic Cages, Blood Gas and Electrolyte Analysis

Mice were placed in metabolic cages (Techniplast, Scanbur, Karlslunde, Denmark) and given three days to acclimatize. Baseline parameters (food intake, water intake, and urine output) as well as urine composition (pH, electrolyte concentration, and osmolality) were determined on day 4. In the metabolic acidosis experiments, the animals were placed in metabolic cages and given 3 days to acclimatize. This was followed by induction of metabolic acidosis by adding 2% NH_4_Cl to the standard chow for 4 days. The collected urine samples were centrifuged at 1000 × *g* for 1 min. Urine pH was measured with a pH-meter (Metrohm, Glostrup, Denmark), whereas ionic composition (Na^+^, K^+^, and Cl^–^) was analyzed at the Medical Research Council Harwell, United Kingdom. Osmolality was measured in 3 × mqH_2_O diluted urine by using a freezing point depression osmometer (model 3320, Advanced Instruments). All urine samples containing feces and larger food products were excluded. The blood samples were collected with heparin-containing PICO syringes (Radiometer, Broenshoej, Denmark) by drawing the blood from the right heart atrium of isoflurane anesthetized mice. The blood gas analysis was performed on FLEX blood gas analyzer (Radiometer).

### Blood Pressure Measurement

Systemic BP was measured non-invasively in 1- to 4-month-old NBCe2^B1–V ATPase cre^ and global NBCe2 ko mice by determining the tail blood volume with a volume pressure recording sensor and an occlusion tail-cuff (CODA System, Kent Scientific, Torrington, CT, United States). Non-anaesthetized mice were placed in a restrainer, and warmed for 5 min on a heating pad. An occlusion cuff and a volume pressure cuff were placed on the tail of the mouse, and the BP was measured. The mice were first placed 10–15 min in the restrainer in order to reduce the stress response to the procedure. In order to further lower the stress response, mice were trained by daily BP measurements for 8–13 days. Measurements were performed at the same time each day in the afternoon when activity in the animal facility was low. The mice were handled by the same researcher each day. There was no statistically significant difference in BP when comparing within the same genotype over duration of the measurement period (not shown). To avoid a systematic bias in the tail cuff equipment, mice were randomly switched between individual cuffs from day to day. One full measurement consisted of 10 acclimation cycles and 10 measurement cycles. Daily values of systolic, diastolic and mean arterial BP were calculated as an average value of the 10 measurement cycles.

### Statistical Analysis

All data is represented as an average ± SEM. Semi-quantification of immunoblotting and NBCe2 ko BP measurements and metabolic cages data were analyzed by an unpaired two-tailed *t*-test and *p* < 0.05 was considered statistically significant.

## Results

### NBCe2 Expression in the Kidney Is Restricted to Cells From CNT and CCD Principal Cells

**FIGURE 1:**
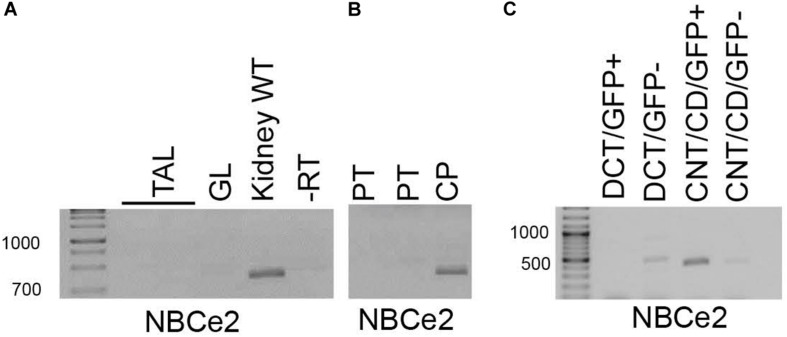
RT-PCR analysis of micro-isolated renal tubules. NBCe2 amplicons were only observed in whole kidney samples and were absent from samples of thick ascending limb (TAL) and glomeruli (GL; **A**). Similarly, a NBCe2 product was absent in samples from proximal tubule (PT) but the present in the positive control, the choroid plexus (CP; **B**). Finally, NBCe2 product was successfully amplified from samples enriched for connecting tubule and collecting duct (CNT/CD) fraction **(C)**. -RT: no reverse transcriptase; DCT-GFP(+); and CNT/CCD-GFP(+): fluorescence positive fraction of FACS sorting, DCT-GFP(–), and CNT/CCD-GFP(–): fluorescence negative fraction of FACS sorting using kidney cells from mice expressing eGFP under control of the parvalbumin (DCT) and TRPv5 (CNT/CCD) promotor, respectively.

NBCe2 expression was determined in isolated mouse kidney tubules and FACS isolated fluorescent tubular cells. Fractions enriched for PT and TAL were collected under a dissection microscope determined by morphology. PT fractions were chosen by the most prominent RT-PCR signal for NBCe1 and AQP1, whereas TAL fractions were chosen by the most prominent signal for NKCC2 and CLC2. DCT fractions were obtained either by morphology or from parvalbumin-promoter driven eGFP expressing mouse tubules. Conversely CNT/CCD fractions were obtained from the branching morphology or from TRPv5-promoter driven eGFP expressing mouse tubules. Although most fractions showed contamination from other tubular segments, NBCe2 expression only coincided with DCT, or CNT/CCD markers. No NBCe2 was detected in TAL and glomeruli in contrast to a whole kidney sample (Figure 1A). Similarly, two PT isolates did not reveal NBCe2 expression in contrast to a choroid plexus sample (Figure 1B). FACS-sorted single cells enriched for CNT/CD showed amplification of a NBCe2 signal (Figure 1C). Weak reaction product was observed in both DCT-GFP(–) cells and TRPv5-GFP(–) cells. As described in Methods, these fractions showed a high level of purity and leave little room for false-positive and false-negative results. The high cycle number necessary to detect NBCe2 in the kidney suggests a relatively low expression level.

### Global NBCe2 Deletion Results in Decreased Base Extrusion From CNT/CCD

In the choroid plexus, NBCe2 exports Na^+^, and HCO_3_^–^ from the epithelial cell across the luminal membrane (Millar and Brown, 2008). We have previously demonstrated that knockout of NBCe2 in the choroid plexus leads to decreased net base extrusion and increased acid extrusion due to the lack of an effective base extruder (Christensen et al., 2018).

In the kidney, the membrane localization of NBCe2 protein in the renal tubules is not known. Using an NBCe2 antibody that specifically recognizes NBCe2 in the choroid plexus, we are unable to determine the subcellular localization of NBCe2 in kidney epithelial cells. We do observe immunoreactivity in PT and DCT similar to what has previously been published by other research groups (Gildea et al., 2015). However, the renal staining is remarkably also present in the kidneys from NBCe2 knockout mice (Supplementary Figure S1). Together, the high cycle number necessary for the detection of NBCe2 by RT-PCR and the lack of detection of renal NBCe2 by immunohistochemistry and immunoblotting suggest a low renal expression level as compared to the choroid plexus.

Similar to the question of subcellular localization, the net transport direction of ions through NBCe2 in the kidney is not known. To investigate the contribution of NBCe2 to base extrusion, intracellular pH was monitored first in either SNARF-loaded or BCECF-loaded isolated tubules (CNT/CCD) from NBCe2 knockout and wild type mice.

**FIGURE 2:**
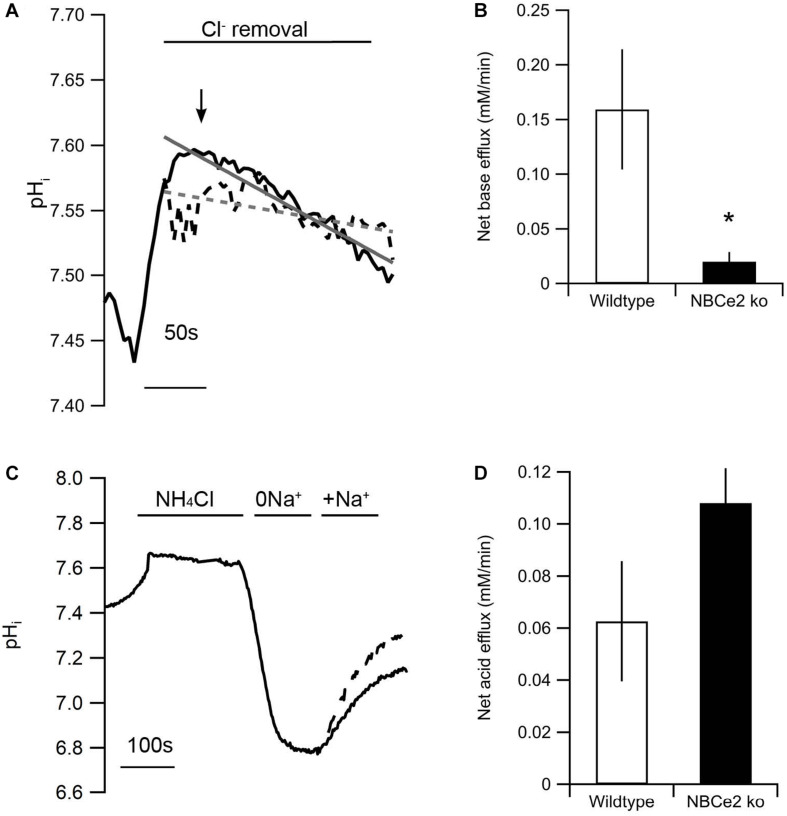
Knockout of NBCe2 results in decreased base extrusion in distal renal tubules. **(A)** Representative traces of intracellular pH (pH_i_) recordings of SNARF loaded isolated distal renal tubules from NBCe2 ko (dotted black line) and wild type mice (full black line). The gray lines show a line fit of the trace from the NBCe2 ko (dotted gray line) and wildtype (full line). Baseline pH_i_ was determined in CO_2_/HCO_3_^–^ buffered saline solution followed by determination of the alkalization induced by removing Cl^–^ in the continued presence of CO_2_/HCO_3_^–^. The rate of base extrusion was determined as the dpH_i_/dt after peak alkalization (arrow). **(B)** Mean values of the net base efflux ± SEM at the peak of Cl^–^ removal. **(C)** Representative traces of pH_i_ recordings of BCECF loaded isolated distal renal tubules from NBCe2 ko (dotted line) and wild type mice (full line). Baseline pH_i_ was determined in a HEPES buffered saline solution. Intracellular pH was decreased by an NH_4_Cl prepulse followed by a washout in Na^+^ free (0Na^+^) solution. The rate of acid extrusion was determined as the dpH_i_/dt after addition of a Na^+^ and HCO_3_^–^ containing solution (+Na^+^). **(D)** Mean values of net acid efflux ± SEM after addition of Na^+^.

Intracellular pH was increased by removal of Cl^–^ from a HCO_3_^–^ buffered solution (Figure 2A). Baseline pH assessed in BBS was not significantly different between the two genotypes [NBCe2 wt: 7.51 ± 0.07 (*n* = 7); NBCe2 ko: 7.56 ± 0.07 (*n* = 6), *p* = 0.64]. The removal of Cl^–^ created a gradient for Cl^–^ out of the cell through the anion exchanger that in return imports HCO_3_^–^ which increases pH_i_. The pH_i_ recovery from this point would be mediated by Cl^–^ independent Na^+^-dependent HCO_3_^–^ *export* (i.e., outward NBC activity). The pH_i_ recovery rate following peak alkalization was decreased by 87% compared to wild type (Figures 2A,B). A different way of assessing NBC activity is to record the Na^+^-dependent HCO_3_^–^ *import* (net acid extrusion) after acidification. The acid extrusion showed a 45% increase in the tubules from NBCe2 knockout but the difference was not statistically significant (Figures 2C,D), but would suggest the lack of NBCe2 mediated HCO_3_^–^ efflux. In order to rule out that the difference in pH_i_ regulation is caused by differences in the buffering capacity of the tubules, the intrinsic buffering capacity was determined. No difference in intrinsic buffering capacity was detected in tubules isolated from knockout and wildtype mice (not shown). Thus, NBCe2 appears to be mediating outward Na^+^:HCO_3_^–^ cotransport in these renal tubules.

### Regulation of Transporters in Kidneys of NBCe2 ko Mice

**FIGURE 3:**
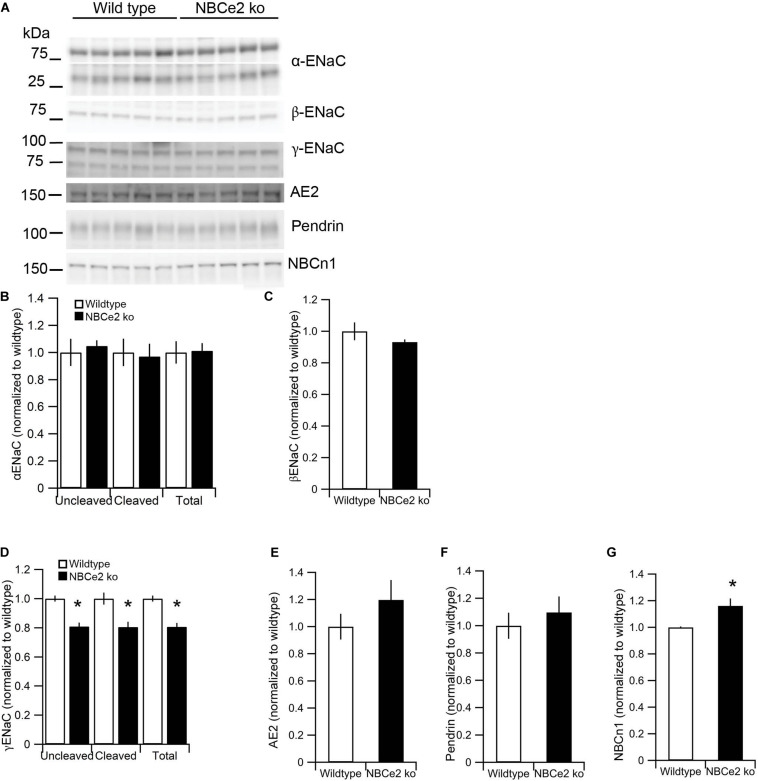
Expression of sodium and bicarbonate transporters in NBCe2 knockout mice. Immunoblotting analysis of kidney protein samples from wildtype (white bars) and NBCe2 global knockout mice (NBCe2 ko, black bars). Blots show immunoreactivity for α, β, and γ-ENaC, NBCn1, pendrin, and AE2 **(A)**. Semi-quantitative analysis of uncleaved (95 kDa band), cleaved (65 kDa band), and total α-ENaC **(B)**, βENaC **(C)** uncleaved (85 kDa band), cleaved (70 kDa band), and total γ-ENaC **(D)**, AE2 **(E)**, pendrin **(F)**, and NBCn1 **(G)**. Data is presented as mean intensity normalized to the control mice ± SEM.

Previous studies have shown differences in expression of the electroneutral Na^+^ and HCO_3_^–^ cotransporter NBCn1 as well as the epithelial sodium channel, ENaC, in kidney from NBCe2 knockout mice. We determined the expression of transporters involved in Na^+^ and HCO_3_^–^ handling in our knockout model. Contrary to previous studies, we found decreased abundance of the γENaC, but no difference in abundance of the α or β subunits (Figures 3A–D). We find increased expression of the Na^+^ and HCO_3_^–^ cotransporter NBCn1 (Figure 3E) but no difference in expression of the Cl^–^/HCO_3_^–^ exchangers pendrin and AE2 (Figures 3F,G).

### NBCe2 Kidney Cell-Specific ko Mice

Since NBCe2 expression and function was only observed in the CNT/CCD, we generated kidney cell specific knockout mice by targeting cells of the CNT and CDs. We have previously shown that NBCe2 is expressed in intercalated cells in the human kidney (Damkier et al., 2007), while previous studies have suggested *slc4a5* mRNA in principal cells (Groger et al., 2012). The NBCe2^B1–VATPasecre^ model expresses cre recombinase under the endogenous *Atp6v1b1* promoter, resulting in recombination in the intercalated cells of the CNT, CD, as well as in 50% of principal cells within the CNT (Miller et al., 2009). We attempted to evaluate the extent of knockout by qPCR but did not obtain a sufficient NBCe2 signal for quantitation. As previously mentioned, a recently characterized NBCe2 specific antibody (Christensen et al., 2018) failed to produce anti-NBCe2 staining in the kidney. The antibody is directed at the N-terminal domain of mouse NBCe2 and should recognize the renal NBCe2 variant. The immunostaining was tested in paraffin sections with target retrieval in high and low pH, as well as in cryostat sections, yet no NBCe2 specific signal was observed. In addition, a proximity ligation assay (PLA, Duolink, Sigma Aldrich) was used in order to amplify the NBCe2 antibody’s binding, but without success apart from the choroid plexus control labeling. Therefore, we were unable to assess the degree of NBCe2 knockout in NBCe2^B1–VATPase cre^ mice.

### Kidney Cell-Specific NBCe2 Deletion Causes Significant Changes in Expression of Renal Membrane Transporters

**FIGURE 4:**
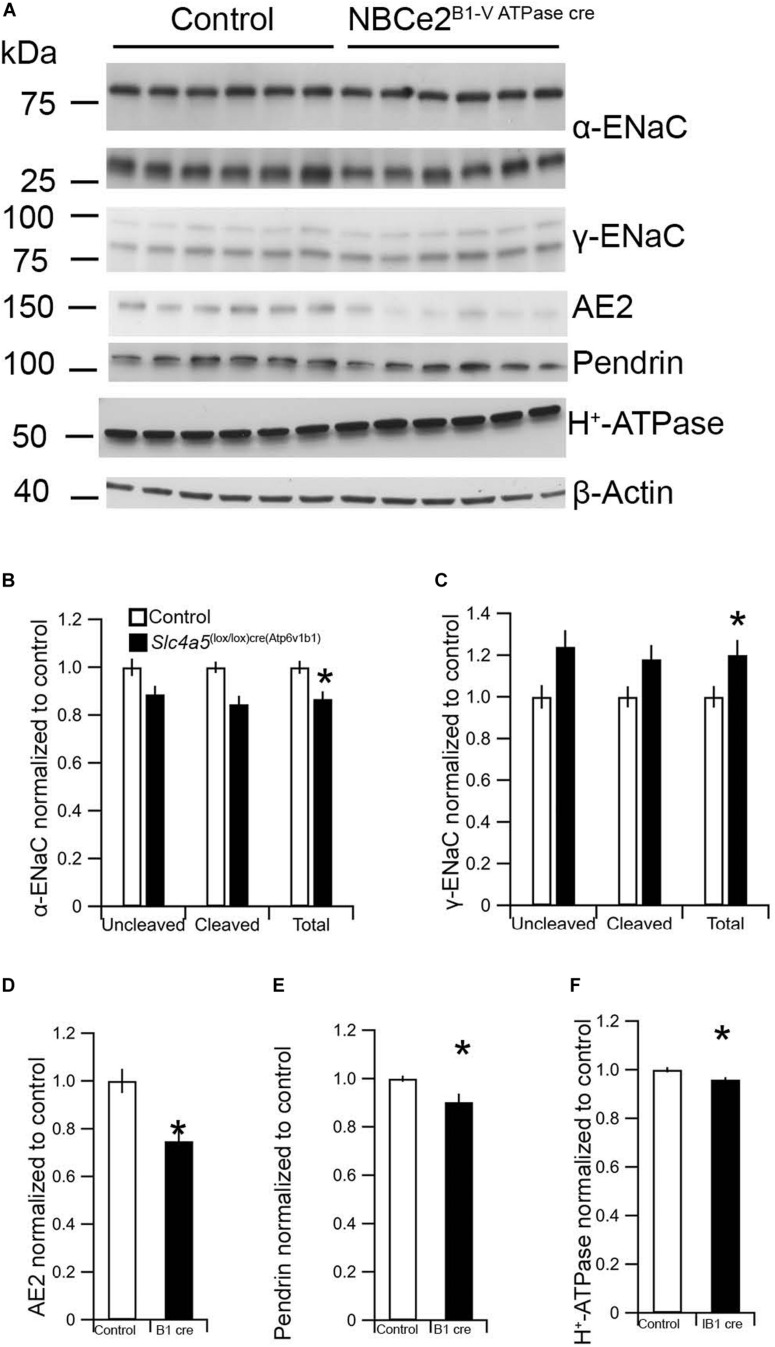
Expression of renal α and β-ENaC as well as H^+^–ATPase in NBCe2^B1–VATPasecre^ mice. Immunoblotting analysis of kidney protein samples from wildtype (white bars) and NBCe2^B1–VATPasecre^ (black bars). Blots show immunoreactivity for α and β-ENaC, AE2, pendrin, H^+^-ATPase, and β-actin **(A)**. Semi-quantitative analysis of uncleaved (95 kDa band), cleaved (65 kDa band), and total α-ENaC relative to β-actin **(B)**; uncleaved (93 kDa band), cleaved (75 kDa band), and total β-ENaC relative to β-actin **(C)**, AE2 **(D)**, pendrin **(E)**, and H^+^-ATPase **(F)** relative to β-actin. Data is presented as mean intensity normalized to the control mice ± SEM.

As it was impossible to evaluate the knockdown degree in the cre model, we evaluated whether the mouse model showed a transporter expression phenotype similar to the global knockout mouse. The expression of some ion transporters, typical for principal and intercalated cells was assessed by semi-quantification from immunoblotting. Unlike the global NBCe2 ko, a significant decrease in cleaved (*p* = 0.005), uncleaved (*p* = 0.04), and total (*p* = 0.011) α-ENaC protein was observed (*n* = 6 for both genotypes) in NBCe2^B1–VATPasecre^ (Figure 4). The uncleaved (*p* = 0.037) and total γ-ENaC (*p* = 0.049) expression was increased at the protein level, whereas the H^+^-ATPase protein expression was decreased (*p* = 0.030) in NBCe2^B1–VATPasecre^. Thus, the NBCe2^B1–VATPasecre^ ko model offers a renal expression pattern separate from that of kidneys from control and global NBCe2 ko mice.

### Kidney Cell-Specific NBCe2 Deletion Causes Minor Alterations in Water and Electrolyte Handling

The baseline renal function was studied in the global NBCe2 ko mice by measuring the basic urine and blood electrolyte parameters. We have previously reported that global NBCe2 ko results in elevated blood pCO_2_ and HCO_3_^–^ (Hofmeister et al., 2009), yet no other parameters statistically differed in this ko model (Table 1).

**TABLE 1:** Baseline parameters in the full NBCe2 ko mice.

Baseline parameters			

	WT	KO	
Food intake, g/day	3.33 ± 0.21	3.29 ± 0.25	n.s.
Water intake, g/day	6.28 ± 0.86	6.14 ± 0.94	n.s.
Urine output (ml/day)	1.41 ± 0.39	1.47 ± 0.37	n.s.
Urine pH	7.50 ± 0.24	7.50 ± 0.20	n.s.
**Plasma parameters**			
Potassium, mmol/l	6.05 ± 0.14	6.18 ± 0.72	n.s.
Blood pH	7.43 ± 0.02	7.43 ± 0.01	n.s.
pCO_2_, kPa (mmHg)	4.4 ± 0.3 (33 ± 2.3)	3.4 ± 0.3 (25.5 ± 2.3)	0.035
StHCO_3__–_, mM	23.1 ± 0.5	20.2 ± 0.7	0.004
Base excess	-2.28 ± 1.12	-4.32 ± 0.85	n.s.
*n*	5	5	

In order to evaluate the cell-type specific NBCe2 knockout effect on renal function, we placed NBCe2^B1–VATPasecre^ mice in metabolic cages to assess baseline water and food intake, urine output and pH, and urine and blood electrolyte composition. NBCe2^B1–VATPasecre^ mice had significantly elevated blood potassium levels with no signs of hemolysis in the samples, whereas the urine potassium level showed a decreaseing tendency (Table 2). NBCe2^B1–VATPasecre^ mice had a numerical but non-statistically significant change in base excess and anion gap in same direction as the global NBCe2 ko.

**TABLE 2:** Baseline blood and urine parameters in the NBCe2^B1–VATPase cre^ mice.

Baseline parameters			

	Control	NBCe2^B1–VATPasecre^	
Mouse weight (g)	25.0 ± 0.1	25.2 ± 0.6	n.s.
Food intake, g/day	5.0 ± 0.7	5.5 ± 0.4	n.s.
Water intake, g/day	4.8 ± 0.4	3.5 ± 0.4	n.s.
Urine output ml/g body weight	0.06 ± 0.02	0.06 ± 0.01	n.s.
**Urine parameters**			
Sodium, mmol/24 h	0.08 ± 0.01	0.12 ± 0.02	n.s.
Potassium, mmol/24 h	0.38 ± 0.01	0.45 ± 0.04	n.s.
Chloride, mmol/24 h	0.22 ± 0.02	0.26 ± 0.02	n.s.
pH	7.37 ± 0.55	7.46 ± 0.17	n.s.
Osmolality (mosm/l)	1990 ± 414	1948 ± 140	n.s.
**Plasma parameters**			
Sodium, mmol/l	147 ± 2	144 ± 1	n.s.
Potassium, mmol/l	6.1 ± 0.3	7.1 ± 0.2	*p* = 0.03
Chloride, mmol/l	110 ± 2	112 ± 1	n.s
Calcium, mmol/l	1.3 ± 0.01	1.3 ± 0.02	n.s.
pH	7.42 ± 0.01	7.45 ± 0.01	n.s.
Hematocrit, %	40 ± 2	33 ± 4	n.s.
Base excess	-0.77 ± 1.22	-1.32 ± 0.91	n.s.
Anion gap	20.4 ± 1.3	15.14 ± 2.8	n.s.
*n*	3	5	

### Increased Blood Pressure in Global NBCe2 Knockout Mice Is Absent in Kidney Cell Targeted ko

**FIGURE 5:**
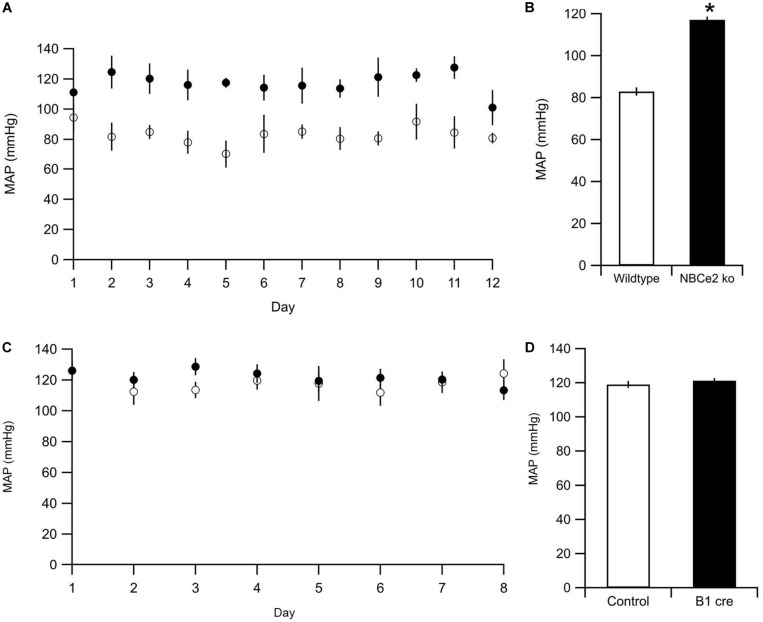
Blood pressure measurements of NBCe2 mice. **(A)** Blood pressure was determined by tail cuff in WT (white dots) and NBCe2 ko mice (black dots). **(B)** Summarized results of mean arterial pressure over the entire 12-day period. **(C)** Blood pressure was determined by tail cuff in control (white dots) and NBCe2^B1–VATPasecre^ (black dots) for eight consecutive days. Dots indicate the mean values ± SEM. **(D)** Summarized results of the mean arterial pressure (MAP) over the entire 8-day period.

To ensure that the tail cuff method is sensitive enough to detect NBCe2 mediated increase in BP, we assessed the BP in NBCe2 ko. As expected, a significantly increased BP was observed in the global NBCe2 ko model (83 ± 1.8 mmHg for wt, 117 ± 7.0 mmHg for ko, *n* = 12 in both groups, *p* = 1.8 × 10^–11^, Figures 5A,B). We then determined the average BP in NBCe2^B1–VATPasecre^ mice by the same method. The mean arterial BP was not increased in the kidney cell-targeted ko model (119 ± 2.0 mmHg for wt, *n* = 9; 120 ± 2.3 mmHg for NBCe2^B1–VATPasecre^, *n* = 8; *p* = 0.569, Figures 5C,D).

## Discussion

In the present study, we show that the renal cortical NBCe2 expression is restricted to the CNT and CD in mice. Exploiting a cell-specific cre mouse model, we targeted deletion of NBCe2 to cells of the CNT/CCD of mouse kidney. The genetic manipulation resulted in altered expression of various sodium and acid-base transporting proteins expressed in the kidney tubules in question. We did, however, not find evidence for increased BP in these mice or changes in basic physiological parameters. This suggests that other organs or cell types are mediating the hypertension observed in the global NBCe2 ko mouse.

We exploited a method for the selection of renal tubules by combining manual tubule collection by morphology, and by FACS sorting of the eGFP expressing tubules. We assessed the purity of the samples by evaluating the expression of tubule segment specific transporters. Previous studies have reported NBCe2 expression in both the proximal part of nephron [PT (Gildea et al., 2015), TAL (Xu et al., 2003)] and in distal nephron and CDs (Damkier et al., 2007; Groger et al., 2012; Wen et al., 2015a), perhaps representing inter-species variation. In the current study, we show that renal cortical NBCe2 is not expressed in the mouse PT, TAL, or DCT, but is only detected in the CNT/CD fractions. Our experiments suggest that it should be a prerequisite to evaluate isolated tubule purity, as only a small fraction of collected samples showed segment-specific expression of transporter mRNA. Although we did not achieve completely pure fractions, we can at least exclude NBCe2 expression in the proximal part of the nephron and confirm its expression in the CNT and CD.

Our functional data indicate that NBCe2 in the CNT/CCD has a similar transport direction as in the choroid plexus: a net outward transport of Na^+^ and HCO_3_^–^. Base extrusion is greatly diminished in alkalized cells and net acid extrusion is numerically increased in acidified cells (however, the latter not significantly). The phenotype could be a result of increased or decreased activity of other acid-base transporters in the cells. In fact, we also found that the abundance of the electroneutral Na^+^;HCO_3_^–^ importer NBCn1 is increased similar to previous publications. NBCn1 is expressed in the basolateral membrane of intercalated cells where it is responsible for the import of HCO_3_^–^ from the blood to the cell (Vorum et al., 2000). The previous study also showed that AE1 is increased in NBCe2 knockout mice (Groger et al., 2012). These two transporters have opposite effects on basolateral base extrusion.

Our RT-PCR results suggest that renal cortical NBCe2 expression is confined to the CNT and CDs. We thus generated a kidney cell specific NBCe2 ko model to further explore the role of NBCe2 by crossing our floxed NBCe2 mouse with a mouse expressing cre recombinase driven by the H^+^-ATPase B1 subunit. This model expresses cre recombinase in all intercalated cells and approximately 50% of the principal cells of the CNT (Ramkumar et al., 2018). Unfortunately, both quantitative PCR and immuno-based techniques failed to detect NBCe2 in the kidney and we were not able to determine the degree of NBCe2 knockdown in the mice. We speculate that the reason why we cannot detect NBCe2 using QPCR and immunohistochemistry is that the abundance of NBCe2 in the kidney is very low. If only a few copies of mRNA are present in the tubules, it would be difficult to get a valid and comparable result with the QPCR from a whole kidney. In comparison, QPCR from the choroid plexus which also expresses NBCe2 gives a stable signal (Christensen et al., 2018). Using RT-PCR of isolated tubules, we are able to detect the level of NBCe2 only with a high cycle number in the PCR. The immunohistochemistry using a previously validated anti-NBCe2 antibody did not detect specific NBCe2 signal in the renal tubules. Increasing the antibody concentration resulted in more background staining and several antigen retrieval methods did not get rid of the background. With a low amount of protein, it would be impossible to detect specific NBCe2 immunoreactivity. In contrast using the same antibody for the choroid plexus revealed intense anti-NBCe2 staining, supporting the notion of a low renal NBCe2 expression level.

Although we cannot estimate how much NBCe2 was excised in our model, we do see changes in the expression of some sodium and acid-base transporters in the kidneys from the NBCe2^B1–VATPasecre^ mouse. This suggests that recombination events have taken place and accounts for the observed expression changes. The cre recombinase in the B1 cre mouse is active in both intercalated cells of the CD as well as principal cells in the CNT (Miller et al., 2009). Previous publications using this cre model has resulted in up to 55–80% reduction in the target protein (Poulsen et al., 2016; Ramkumar et al., 2018). The fact that the NBCe2^B1–VATPasecre^ mouse presents with a phenotype suggests that functionally relevant NBCe2 could be expressed in the intercalated cells as well as in principal cells of the CNT.

The NBCe2^B1–VATPasecre^ mice show a different expression pattern of transporters compared to the global knockout. The α-ENaC expression is decreased, whereas uncleaved and total γ-ENaC are increased. The full knockouts, however, show decreased expression of γ-ENaC but normal levels of the α and β subunits of ENaC. In the previously reported NBCe2 knockout mice α-ENaC was found to be increased. The discrepancy between the two knockout mice could be explained by differences in the knockout strategy or differences in animal housing (e.g., sodium content in the chow) in the laboratories.

Additionally in the NBCe2^B1–VATPasecre^ mouse, we observed a significant decrease in V-ATPase expression, which is opposite to the effect observed in previously published full NBCe2 ko mice on an acidic diet (Wen et al., 2015a) where an increased expression is observed. We also found a decreased abundance of two of the anion exchangers, AE2 and pendrin. Both of these are found to be expressed at normal levels in the global NBCe2 knockout mouse. The difference in expression pattern of other proteins could potentially explain the lack of a BP phenotype increase in the NBCe2^B1–VATPasecre^. For example, a decrease in pendrin could potentially have an opposing effect on BP (López-Cayuqueo et al., 2018). Ablation of pendrin is known to result in lowering of BP (Trepiccione et al., 2017) as pendrin is known to affect ENaC mediated by changing urinary HCO_3_ (Pech et al., 2010). We cannot explain the discrepancy in pendrin expression in the full knockout and the NBCe2^B1–VATPasecre^ mouse. The possible interplay between NBCe2 expression in principal cells of the CNT and pendrin in the intercalated cells needs to be further investigated.

The metabolic cage data showed only minor changes in water and electrolyte parameters. We observed increased plasma K^+^ in the NBCe2^B1–VATPasecre^ model, which is opposite to what was seen in the study by Wen et al. (2015a). Stimulation with aldosterone or increased ENaC activity in the principal cells usually results in potassium wasting, as increased sodium uptake via apical ENaC channels stimulates basolateral Na^+^ excretion into the blood in exchange for K^+^. We observe the opposite effect, i.e., an increase in plasma K^+^ and a decreased cleaved α-ENaC expression in the global NBCe2 ko model, which might suggest a reduction of ENaC activity as a response to the increase in BP. No significant differences were observed during baseline metabolic assessment in the global NBCe2 ko model, apart from the previously reported decrease in blood pCO_2_ and HCO_3_^–^.

In accordance to previous studies, full NBCe2 ko resulted in significantly increased BP, as measured by the tail-cuff method, whereas kidney-targeted NBCe2 ko did not present with this trait. We did not assess the BP in our kidney ko models on an acidic diet. Although the BP assessment by tail-cuff is not as accurate as telemetric measurements, the difference between the full NBCe2 ko and kidney cell-targeted NBCe2 ko is clearly evident.

It is possible that the discrepancy between the full ko and the B1-cre model is caused by insufficient knockdown of NBCe2 in the kidney. This would result in a different phenotype than that of a global NBCe2 knockout. Another explanation is that dysfunctional kidney NBCe2 might not be the only cause of the increased BP observed in full NBCe2 ko, as NBCe2 expression is close to undetectable in the mouse kidney, and kidney cell targeted NBCe2 ko had no apparent BP phenotype. In addition to the kidney, NBCe2 is expressed in the liver and in especially high abundance in the choroid plexus of the brain. The choroid plexus secretes the majority of the cerebrospinal fluid. Changes in cerebrospinal fluid electrolytes have previously been shown to affect the central regulation of BP through activation of the sympathetic nervous system. While this study clearly shows that the renal NBCe2 may play a role in local regulation of HCO_3_^–^ in the kidney tubule cells, further investigations are necessary to understand the pathogenesis and possible treatment of this particular cause of hypertension in relation to dysfunctional NBCe2.

In conclusion, renal NBCe2 expression seems to be confined to CNT and CD, and thus, we cannot confirm previous reports of NBCe2 expression in proximal or distal tubules. In isolated CNT and CCD, NBCe2 mediates outward Na^+^-dependent HCO_3_^–^ transport from either the basolateral or luminal membrane. Targeted knockout of NBCe2 in intercalated cells led to a different pattern of changes in renal transport protein expression levels than observed in global NBCe2 knockout mice. The targeted knockout of NBCe2 in CNT/CCD did not lead to the hypertensive phenotype observed in the global NBCe2 knockout. Thus, we speculate that the BP elevation in NBCe2 knockout mice does not arise from lack of NBCe2 expression in the CNT and CD, but indicate that other cell types or potentially other organ systems are involved in the NBCe2 dependent BP regulation.

## Data Availability Statement

The datasets generated for this study are available on request to the corresponding author.

## Ethics Statement

The animal study was reviewed and approved by The Danish Animal Experiments Inspectorate.

## Author Contributions

HD and JP contributed to the conception and design of the work. DB, FP, HC, LJ, and HD contributed to the acquisition of the data. All authors contributed to the analysis and interpretation of the data for the manuscript. DB drafted the initial manuscript and HD and JP revised it critically for important intellectual content. All authors contributed to manuscript revision, read and approved the submitted version.

## Conflict of Interest

The authors declare that the research was conducted in the absence of any commercial or financial relationships that could be construed as a potential conflict of interest.
