# Supplementary figures and images for: NBCe2 (Slc4a5) Is Expressed in the Renal Connecting Tubules and Cortical Collecting Ducts and Mediates Base Extrusion
Source: Front Physiol. 2020 May 29;11:560. doi: 10.3389/fphys.2020.00560 (PMC7273925; doi:10.3389/fphys.2020.00560)

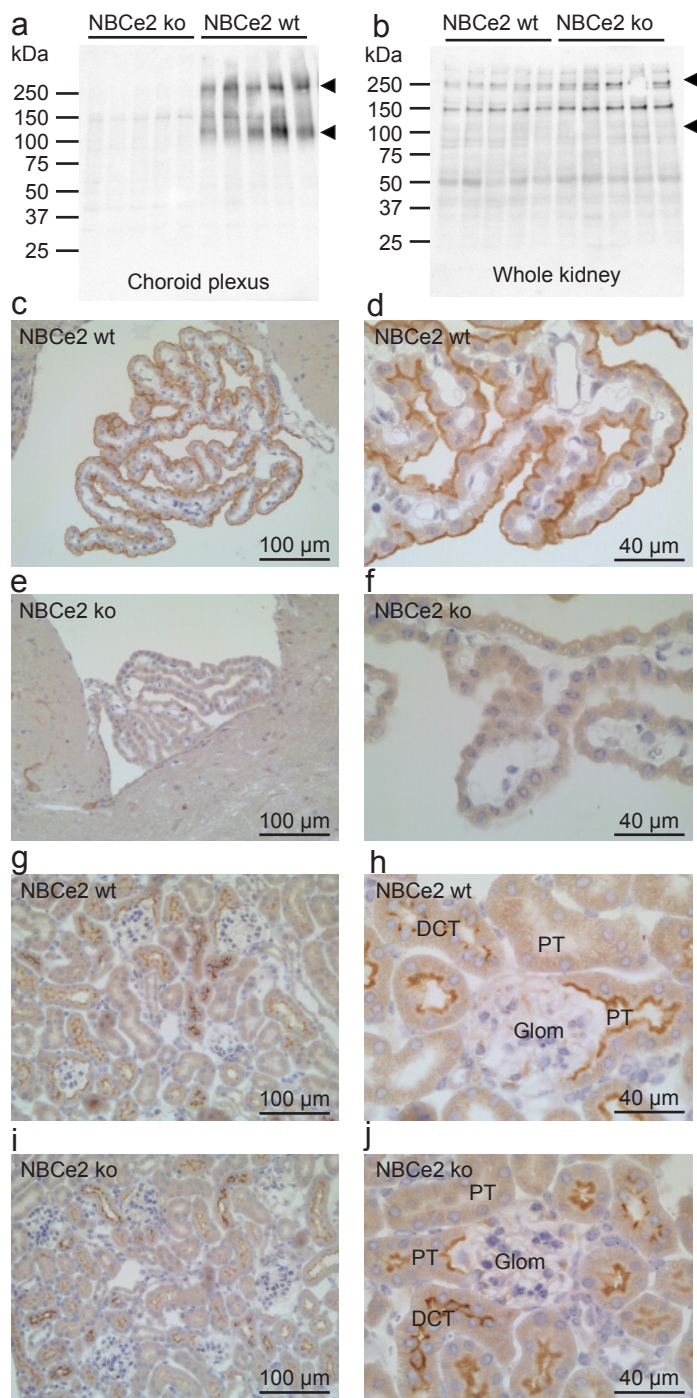

Supplemental figure S1

Supplement: Supplementary file 1 [file Data_Sheet_1.PDF]
