# Supplementary material for: NBCe2 (Slc4a5) Is Expressed in the Renal Connecting Tubules and Cortical Collecting Ducts and Mediates Base Extrusion
Source: Front Physiol. 2020 May 29;11:560. doi: 10.3389/fphys.2020.00560 (PMC7273925; doi:10.3389/fphys.2020.00560)
Supplement: Supplementary file 2 [file Table_1.DOCX]

Supplementary material

NBCe2 (*slc4a5)* is expressed in the renal connecting tubules and cortical collecting ducts and mediates base extrusion

**Supplementary methods**

***Immunohistochemistry***

Mice were perfusion fixed through the heart with 4% paraformaldehyde in PBS. Following perfusion-fixation the brain and kidneys were removed and post-fixed for 2 hours, dehydrated and embedded in paraffin wax. The tissue was cut into 2 µm sections using a rotary microtome (Leica, Wetzlar, Germany). The sections were dewaxed and rehydrated followed by retrieval of the epitopes by boiling the sections in a 10 mM Tris-buffer (pH 9) with 0.5 mM EGTA. Aldehydes were reduced with 50 mM NH_4_Cl in PBS and unspecific binding blocked by washing with a 1% BSA solution in PBS with 0.2 % gelatin and 0.05% saponin. The sections were incubated overnight with the primary antibody diluted in 0.1% BSA in PBS added 0.3% Triton X-100 at 4°C. The sections were incubated with horseradish peroxidase-conjugated goat anti-rabbit IgG (DAKO) in PBS with BSA and Triton X-100 and the staining was visualized by 0.05% 3,3’-diaminobenzidine tetrahydrochloride dissolved in PBS with 0.1% H_2_O_2_. Mayer’s hematoxylin was used for counterstaining, and the sections were dehydrated in graded alcohol and xylene and mounted in Eukitt mounting medium. Microscopy was performed on a Leica DMRE brightfield microscope with a Leica DM300 digital camera.

**Supplementary tables**

**Table S1.** **Primers used for RT-PCR.**

| **Transcript** | **Tubule localization** | **Direction** | **Sequence** | **Product size** |
| --- | --- | --- | --- | --- |
| NBCe1 | PT | Sense | ctcacttctcctgtgcttgcct | 297 |
|  |  | Antisense | gtggttggaaaatagcggctgg |  |
| NKCC2 | TAL | Sense | tcctgaccaagaaccttcctcc | 500 |
|  |  | Antisense | tagccacctgccttcaccatac |  |
| NCC | DCT | Sense | ttcccgacatcaaccagaagcc | 226 |
|  |  | Antisense | agcagcatcccgagagtaatcc |  |
| AQP1 | PT | Sense | ggccatgaccctcttcgtct | 198 |
|  |  | Antisense | gagcagGagccccagtgtga |  |
| CLC2 | TAL, mTAL | Sense | agagtacctcaccctcaagacc | 359 |
|  |  | Antisense | atcacggttccacactgccaag |  |
| AQP2 | CNT, CD | Sense | Tggctgtcaatgctctccac | 199 |
|  |  | Antisense | ggagcagccggtgaaataga |  |
| Actin | All | Sense | acatggcattgttaccaactgg | 880 |
|  |  | Antisense | cggactcatcgtactcctgctt |  |
| NBCe2 |  | Sense | tctgggggatgccacagacaac | 423 |
|  |  | Antisense | agcgacgcactcgcatttgtag |  |

**Table S2. Salt solutions for live cell fluorescence microscopy**

| **Substance (mM)** | **HBS** | **NH_4_Cl HBS** | **Na^+^ free BBS** | **BBS** | **Cl^-^ free BBS** | **High K^+^ HBS** |
| --- | --- | --- | --- | --- | --- | --- |
| Na^+^ | 145 | 125 | 0 | 145 | 145 | 10 |
| K^+^ | 3.6 | 3.6 | 3.6 | 3.6 | 3.6 | 138.6 |
| Ca^2+^ | 1.8 | 1.8 | 1.8 | 1.8 | 1.8 | 1.8 |
| Mg^2+^ | 0.8 | 0.8 | 0.8 | 0.8 | 0.8 | 0.8 |
| NH_4_^+^ |  | 20 |  |  |  |  |
| Cl^-^ | 138.6 | 138.6 | 138.6 | 138.6 |  | 138.6 |
| SO_4_^-^ | 0.8 | 0.8 | 0.8 | 0.8 | 0.8 | 0.8 |
| HCO_3_^-^ |  |  | 24 | 24 | 24 |  |
| Glucose | 5.5 | 5.5 | 5.5 | 5.5 | 5.5 | 5.5 |
| HEPES | 10 | 10 | 10 | 10 | 10 | 10 |
| NMDG |  |  | 121 |  |  |  |
| Choline |  |  | 24 |  |  |  |
| PO_4_^3-^ | 2 | 2 | 2 | 2 | 2 | 2 |
| mOsm | 308 | 308 | 308 | 308 | 308 | 308 |
| pH | 7.4 | 7.4 | 7.4 | 7.4 | 7.4 | 7.4 |
| CO_2_ |  |  | 5% | 5% | 5% |  |

HBS: HEPES-buffered solution; BBS: CO_2_/HCO_3_^-^ buffered solution; HEPES: (4-(2-hydroxyethyl)-1-piperazinetanesulfonic acid; NMDG: N-methyl-D-glucamine

**Supplementary figure legends**

**Supplementary figure 1S. Expression of NBCe2 in mouse kidney.** The anti-NBCe2 antibody recognizes a band of 126 kDa as well as an app. 250 kDa band corresponding to the dimerized protein. In the choroid plexus (a), the bands are present in the wildtype mice (NBCe2 wt) and absent in the NBCe2 knockout mouse (NBCe2 ko). In the kidneys (b), however, there are bands at the corresponding sizes but these bands are equally present in wildtype and NBCe2 knockout mice and are most likely unspecific binding. In the choroid plexus immunoreactivity using the anti-NBCe2 antibody is observed in the luminal membrane domain of NBCe2 wt (c and d) and absent in the NBCe2 ko (e and f). In the kidney, immunoreactivity is observed in the proximal tubule (PT) as well as the distal convoluted tubules of both the NBCe2 wt (g and h) and the NBCe2 ko (i and j) indicating that the labelling pattern is not specific to NBCe2.
